# Supplementary material for: Monitoring Training Load, Well-Being, Heart Rate Variability, and Competitive Performance of a Functional-Fitness Female Athlete: A Case Study
Source: Sports (Basel). 2019 Feb 9;7(2):35. doi: 10.3390/sports7020035 (PMC6409702; doi:10.3390/sports7020035)
Supplement: Supplementary file 1 [file sports-07-00035-s001.pdf]

Supplementary Materials

Example of a weekly training program

	Monday	Tuesday	Wednesday	Thursday	Friday	Saturday	Sunday
Strength	A1. Every 2 minutes, for 8 minutes (4 sets): snatch Press from Receiving x 2 reps<br>A2. Every 2 minutes, for 6 minutes (3 sets): muscle Snatch x 2 reps<br>A3. Every 2 minutes, for 6 minutes (3 sets): drop Snatch x 2 reps<br><br>B. For time: 20 snatches @ 80% of 1-RM<br><br>C. Five sets of: front squat x 3 reps @ 85% of 1-RM<br><br>D. Three sets of: GHD weighted hip extension x 12 reps; bulgarian split squats x 8 reps each leg	A. Every 2 minutes, for 8 minutes (4 sets):<br>Sotts Press x 3 reps<br><br>B. Take 20 minutes to build to today's 1-RM: Power Clean + Power Jerk<br><br>C. Every 2 minutes, for 20 minutes (10 sets): Split Jerk<br>*Sets 1-2 = 3 reps @ 65%<br>*Sets 3-4 = 3 reps @ 70%<br>*Sets 5-6 = 2 reps @ 75%<br>*Sets 7-8 = 2 reps @ 80%<br>*Sets 9-10 = 1 rep @ 85%	A. Three sets of:<br>Pause Deadlifts x 8 reps @ 62.5%<br>Rest 3 minutes<br><br>B. Three sets of:<br>Dumbbell Floor Press x 8 reps<br>Rest 2 minutes<br><br>C. Four sets of:<br>Dumbbell Skull Crushers x 10 reps<br>Rest 2 minutes<br><br>D. Three sets of:<br>Back Squat x 8 reps @ 72.5%<br>Rest 3 minutes	Rest day	A1. Every 2 minutes, for 6 minutes (3 sets) of:<br>Snatch Press from Receiving x 3 reps<br>A2. Every 2 minutes, for 6 minutes (3 sets) of:<br>Drop Snatch x 1 rep<br><br>B. Build to today's heavy Snatch from Hang<br>Every 90 seconds, for 6 minutes (4 sets):<br>Snatch x 1 rep<br><br>C. Front Squat<br>*Set 1 – 4 reps @ 65-70%<br>*Set 2 – 3 reps @ 70-75%<br>*Set 3 – 2 reps @ 75-80%<br>*Set 4 – 2 reps @ 80-85%<br>*Set 5 – 1 rep @ 85-90%<br>*Set 6 – 1 rep @ 90%<br>*Set 7 – 1 rep @ 92-95%	A. Build to today's 1-RM Clean & Jerk<br><br>B. Every 90 seconds, for 6 minutes (4 sets):<br>Clean & Jerk x 1 rep @ 90% of today's 1RM<br><br>C. Three sets of:<br>Split Stance Romanian Deadlift x 8 reps each leg<br>Rest 60 seconds<br>Kettlebell Biceps Curls x 8 reps<br>Rest 60 seconds	Rest day
Conditioning	Every 10 minutes, for 30 minutes (3 sets):<br>20 sandbag squats (150/100 lbs)<br>400 m run<br>10 muscle-ups<br>20 sandbag squats (150/100 lbs)<br>400 m run	For time:<br>30 Chest-to-Bar Pull-Ups<br>6 Ground to Overhead (245/165 lbs)<br>20 Chest-to-Bar Pull-Ups<br>4 Ground to Overhead<br>10 Chest-to-Bar Pull-Ups<br>2 Ground to Overhead	A. Three rounds for time of:<br>15 Dumbbell Thrusters (50/35 lbs)<br>12 Toes-to-Bar<br>9 Burpees Over the Dumbbells<br><br>B. For time:<br>100 Double-Unders<br>500 Meter Row<br>50 Alternating Pistols<br>500 Meter Row<br>100 Double-Unders		Four sets for times of:<br>10 Muscle-Ups<br>20 Box Jump-Overs<br>30 Dumbbell Snatches (50/35 lbs)<br>400 Meter Run<br>Rest 4 minutes	Every minute, on the minute, for 21 minutes:<br>Minute 1: 8 Burpee Dumbbell Thrusters (35/25 lbs)<br>Minute 2: 15/10 Calorie Assault Bike<br>Minute 3: 15 Pull-Ups	

Accessory	Strength Accessory Option<br><br>A. Every minute, on the minute, for 7 minutes:<br>5 supinated grip bent over barbell rows<br>10 second max effort assault bike sprint<br><br>B. Three sets of:<br>sandbag squat x max reps<br>100 meter sled push<br><br>C. Three sets of:<br>harnessed bear crawl x 30 m	Strength Accessory Option<br><br>A. Three sets of:<br>EZ-Bar Bicep Curl x 10 reps<br>Rest 30 seconds<br>EZ-Bar Tricep Extension x 10 reps<br>Rest 60 seconds<br><br>B. Three sets of:<br>Hanging Straight Leg Raises x AMRAP<br>Rest 90 seconds<br>Extended Plank x 60 seconds<br>Med Ball Slams x 20 second AMRAP	Strength Accessory Option<br><br>Three sets of:<br>Double Kettlebell Romanian Deadlift x 12 reps<br>Rest as needed<br>Single-Leg Barbell Glute Bridge x 10 reps each leg<br>Rest as needed	Strength Accessory Option<br><br>A. Two sets for max reps of:<br>60 seconds of Stiff Leg Deadlift @ 25% of 1-RM Deadlift<br>Rest 60 seconds<br><br>B. In 12 minutes build to today's 3-RM Sumo Deadlift<br><br>C. Two sets of:<br>Deadlift x Max Reps @ 75% of 1-RM<br>Heavy Sandbag Carry for max distance<br>Rest 90 seconds<br><br>D. One set of:<br>200 Meter Sled Sprint	Strength Accessory Option<br><br>A. Three sets of:<br>Supinated-Grip Chinese Barbell Rows x 8-10 reps<br>Rest as needed<br><br>B. Three Sets of:<br>Half-Kneeling Landmine Rotations (right side) x 10 reps<br>Rest 30 seconds<br>Right Side Plank x 30 seconds<br>Rest 60 seconds<br>Half-Kneeling Landmine Rotations (left side) x 10 reps<br>Rest 30 seconds<br>Left Side Plank x 30 seconds<br>Rest 60 seconds
Gymnastics		Aerobic/Gymnastics Option<br><br>Every 8 minutes, for 24 minutes (3 sets) of:<br>40/30 Calorie Assault Bike<br>5 Bar Muscle Ups<br>10 Strict Handstand Push Ups to 10/5 cm Deficit<br>5 Bar Muscle Ups	Strict Gymnastics Volume Accumulation Option<br><br>Six sets of:<br>Ring Support Hold x 20 seconds<br>Rest 15 seconds<br>Strict Toes to Bar x 10 reps<br>Rest 15 seconds<br>Ring Dip Hold x 20 Seconds<br>Rest 15 seconds<br>Strict Pull-Ups x Max Reps<br>Rest as needed	Four sets of:<br>Supinated-Grip Strict L-Pull Ups x 3-5 reps<br>Rest 60 seconds<br>Handstand Walk x 30mts<br>Rest 60 seconds<br>Rope Climb x 2 ascents<br>Rest as needed	
Endurance	Assault Bike Conditioning Option:<br>Every 2 minutes, for 14 minutes (7 sets) of:<br>25 Calories of Assault Bike		Six sets for times of:<br>60 seconds max Calorie Ski-Erg<br>60 seconds max Calorie Row	Assault Bike Conditioning Option<br>Two sets of:<br>10 minutes of Assault Bike<br>Rest 5 Minutes	Running Endurance Option<br>Four sets for times of:<br>Run 2000 Meters @ 80-85% of 1-Mile Pace<br>Rest 2 minutes

Running Endurance Option<br>Six sets of:<br>Run 200 Meters @ 100% of 1-Mile Pace<br>Rest 60 seconds<br>Run 200 Meters @ 100% of 1-Mile Pace<br>Rest 60 seconds<br>Run 400 Meters @ 80% of 1-Mile Pace<br>Rest 60 seconds	60 seconds max Calorie Assault Bike<br>60 seconds Sand Bag Bear Hug Hold (HEAVY)<br>Rest 2 minutes	Rowing Endurance Option<br>Two sets for times of:<br>Row 4000 Meters<br>Rest 4 minutes
